# P05.30. Integrating complementary and alternative medicine into mainstream health care: an empirical study of seven health care services in Australia

**DOI:** 10.1186/1472-6882-12-S1-P390

**Published:** 2012-06-12

**Authors:** J Singer, J Adams, J Adams

**Affiliations:** 1 University Centre for Rural Health, Sydney University, Lismore, Australia; 2 Faculty of Nursing, Midwifery and Health, University of Technology Syd, Sydney, Australia; 3 Victorian Foundation for Survivors of Torture, Melbourne, Australia

## Purpose

To date, most studies of integrative health care (IHC) have focused on the experiences of patients and practitioners, often emphasising the tensions between CAM and biomedical cultures. Minimal research has investigated the perspectives of IHC managers. In response, this study explores the perspectives of seven IHC managers working in a diverse range of health care services in Australia, in which CAM has been incorporated as part of service delivery. The services comprised: five community-based programs including drug and alcohol rehabilitation, refugee mental health, women’s health, and two hospital-based specialist services dealing with chronic conditions. The CAM practices included acupuncture, naturopathy, western herbal medicine and massage amongst others.

## Methods

Using in-depth interviews, this exploratory study examined the perceptions of clinical managers about the role of CAM in their services and the models and strategies employed for integrating CAM into clinical care. Key informant interviews were also conducted with a CAM academic, a CAM practitioner and a palliative care physician.

## Results

Preliminary findings indicate that the managers perceive CAM as providing greater health care choice; help deliver ‘more holistic’ services; filling a therapeutic gap; enhancing quality of life; and providing clients with a ‘point of entry’ which enables them to access biomedical and psychological treatments.

## Conclusion

Preliminary findings from this qualitative study suggest a set of positive examples of integrative health care in which biomedical and CAM practitioners work collaboratively. Our findings also provide exemplars of health care contexts in which CAM and psychological therapies collaborate to provide innovative approaches for the treatment of trauma. Through the perspectives of managers in a range of different integrative clinical settings, this study is able to further our understandings about the practice of IHC.

